# [Retracted] IQGAP1 plays an important role in the cell proliferation of multiple myeloma via the MAP kinase (ERK) pathway

**DOI:** 10.3892/or.2025.8882

**Published:** 2025-03-04

**Authors:** Yongyong Ma, Zhouxiang Jin, Jin Huang, Shujuan Zhou, Haige Ye, Songfu Jiang, Kang Yu

Oncol Rep 30: 3032–3038, 2013; DOI: 10.3892/or.2013.2785

Following the publication of the above paper, a concerned reader drew to the Editor's attention that certain discrete western blot data shown in the RT-qPCR blots in Figs. 1A and 2A, and western blot data in Fig. 5 were strikingly similar to data that had already appeared in previously published papers in different journals written by different authors at different research institutes. Furthermore, some of the protein bands featured in the western blots in Figs. 1C, 2C and 4 were strikingly similar to other bands within the same figures, suggesting that the figures in question may have undergone inappropriate editing.

Having considered the issues identified, the Editor of *Oncology Reports* has decided that this article should be retracted from the Journal on account of the re-use of previously published data and the uncertainties regarding the presentation of data in Figs. 1C, 2C and 4. The authors were asked for an explanation to account for these concerns, but the Editorial Office did not receive a satisfactory reply. The Editor apologizes to the readership for any inconvenience caused.

